# Impact of Job Insecurity on Psychological Well- and Ill-Being among High Performance Coaches

**DOI:** 10.3390/ijerph17196939

**Published:** 2020-09-23

**Authors:** Marte Bentzen, Göran Kenttä, Anne Richter, Pierre-Nicolas Lemyre

**Affiliations:** 1 Department of Teacher Education and Outdoor Life Studies, Norwegian School of Sport Sciences, 0806 Oslo, Norway; 2 School of Business, University of South-Eastern Norway, 3045 Drammen, Norway; 3 Department of Performance and Training, The Swedish School of Sport and Health Sciences, 11433 Stockholm, Sweden; Goran.Kentta@gih.se (G.K.); anne.richter@ki.se (A.R.); 4 The School of Human Kinetics, University of Ottawa, Ottawa, ON K1N 6N5, Canada; 5 Karolinska Institute, 17177 Stockholm, Sweden; 6 Department of Sport and Social Science, Norwegian School of Sport Sciences, 0806 Oslo, Norway; nicolasl@nih.no

**Keywords:** high performance coaches, job insecurity, values, psychological health

## Abstract

Background: The evaluative nature of high performance (HP) sport fosters performance expectations that can be associated with harsh scrutiny, criticism, and job insecurity. In this context, (HP) sport is described as a highly competitive, complex, and turbulent work environment. The aim of this longitudinal, quantitative study was to explore whether HP coaches’ perceptions of job insecurity and job value incongruence in relation to work would predict their psychological well- and ill-being over time. Methods: HP coaches (*n* = 299) responded to an electronic questionnaire at the start, middle, and end of a competitive season, designed to measure the following: job insecurity, values, psychological well-being (vitality and satisfaction with work), and psychological ill-being (exhaustion and cynicism). Structural equation model analyses were conducted using Mplus. Results: Experiencing higher levels of job insecurity during the middle of the season significantly predicted an increase in coaches’ psychological ill-being, and a decrease in their psychological well-being at the end of the season. However, value incongruence did not have a significant longitudinal impact. Conclusions: These findings cumulatively indicate that coaches’ perceptions of job insecurity matter to their psychological health at work. Consequently, it is recommended that coaches and organizations acknowledge and discuss how to handle job security within the HP sport context.

## 1. Introduction

Stress is, and will always be, an integral part of competitive sport. Importantly, coaching has the potential to be rewarding and not everyone that experiences demands and stressors will suffer [1]. Nevertheless, the high performance (HP) sport work environment is considered to be highly demanding, unpredictable, and insecure [2]. High performance coaches (HPCs) report a combination of frequently occurring stressors within the personal, organizational, and competitive domains [3,4]. Consequently, it has been suggested that being an HPC is an all-consuming and demanding profession without clear boundaries, which can ultimately be detrimental to their mental health [4,5,6]. The increasingly competitive and evaluative nature of elite sport fosters performance expectations that are associated with harsh scrutiny, criticism, and job insecurity [7]. Typically, demands accumulate from constant performance pressure, an extensive travel schedule, long and irregular work hours, team selections, decisions, and multiple roles and responsibilities [8,9]. Altogether, these stressors and demands can lead to various negative consequences for coaches’ psychological health, such as work-home interference, a sense of isolation in the coaching role, and psychophysiological outcomes such as sleep disturbance, changes in mood, and burnout [5,10,11,12,13].

### 1.1. Psychological Health and Job Demands

Mental health has recently become an important topic in sport science, as evidenced by the publication of five position statements during 2018–2019 [14,15,16,17,18]. However, most of the attention in these articles was directed towards athletes. Moreover, issues related to poor mental health among coaches have so far been limited to burnout and maladaptive stress-related symptoms [5,19]. It is noteworthy that these negative outcomes are related to the concepts of ill-being and mental illness [20,21], which represent the darker side of mental health. In contrast, and just as important, is the brighter side of mental health, consisting of both positive mental health and a lack of mental illness. More specifically, mental health, as something distinctly separate from mental illness, could be viewed as a continuum from having low positive mental health to high positive mental health [20,21]. In order to more fully understand mental health in sport as a complex, dynamic, and comprehensive concept, there is a need for greater awareness and knowledge of both ill-being and well-being. However, in a recent review on stressors, coping and well-being among sport coaches, only five studies that focused on coaches’ well-being were identified [8]. While acknowledging that HPCs work in a demanding context, there is a need for more specific studies of the factors that could explain why some coaches experience either well-being or ill-being over time at work [22]. 

The job demands-resources (JD-R) model is a well-established framework within occupational psychology that explains how different occupations have their own specific factors related to job stress, namely job resource and job demands [23]. Job resources refer to those physical, social, or organizational aspects of work that reduce job demands, which are associated with psychological costs, and/or stimulate personal growth, learning, and development [23]. For HP coaches, this could be leader support and autonomy in their job. Within the revised JD-R model, job demands have been differentiated into two qualitatively different subcategories, namely job challenges and job hindrances [24,25]. Job challenges are defined as demands that require energy to be solved, and therefore can be a strain on the employee. However, challenges also have the potential to be energizing and stimulating with the potential for problem solving, which can lead to goal attainment, as well as pleasant and fulfilling experiences [24]. For HP coaches this could be systematically striving in their coaching to bring out the best potential in each available athlete on the team. Job hindrances are demands that are experienced as constraining the employee and are related to emotional and cognitive burdens. These types of job demands, which are positively related to ill-being, are often outside the employees’ control and, consequently, more likely to hinder optimal functioning [24]. For HP coaches, this could, for instance, be restricted finances of the sports club, interpersonal conflicts, or chronic injuries of important athletes. 

Job insecurity as a job stressor has received considerable research attention in work and organizational psychology and has been acknowledged to be an inherent part of the job as an HPC [4,8,9]. Furthermore, job value incongruence has been identified to be a stressor in work settings [26], however, it is not frequently applied to HPCs. Hence, the goal of this study was to examine how job value incongruence and job insecurity are related to HPCs’ well- and ill-being over time.

### 1.2. Values in Coaching

Within sport sciences literature, coaching philosophy is a broad research topic, which includes values, beliefs, and principles. According to Burton and Raedeke [27], coaching philosophy is a set of beliefs and principles that guide coaches’ behaviors. The philosophy helps coaches to remain true to their values, while handling the hundreds of coaching choices they must make. Values have been defined as “an enduring belief that a particular way of behaving or living is personally and socially preferable to other ways of behaving and living” [28] (p. 56). Coaches’ actions are either consciously, or unconsciously, influenced by their values [29]. Although values arguably guiding coaches’ work, there is a lack of research focusing solely on values among coaches, and a dearth of research specifically addressing how values are developed and linked to coaching actions [30]. Within work and organizational psychology, values have been studied in relation to how well- and ill-being is affected when there is an (in)congruence between one’s values and behaviors [31]. When employees perceive that their own personal values are in line with the organization’s core mission, a match is experienced [32]. This fit has been shown to be important for the employees’ psychological health [33]. Moreover, when organizational and personal values are perceived to be congruent, this leads to higher levels of motivation, initiative, energy, and efficacy at work [34,35]. Following this line of thought, value congruence has been operationalized as having the potential to be a job resource within the framework of JD-R [36]. In contrast, distress and tension occur when a mismatch in values is experienced, which has been identified to play a central role in the development of burnout [35,37]. Mismatches in values can be evident from the beginning of an employment relationship and can also develop over time due to maturation in relationships or changes in circumstances [32]. Subsequently, experiencing value incongruence at work could also be understood as a job hindrance within the JD-R model [24]. In a study among HPC, the perception of job value incongruence was found to play a crucial role in the erosion of motivation and the exhaustion process [38]. Nevertheless, existing literature that explores the longitudinal consequences for coaches’ psychological well- and ill-being when acting in (in)congruence with their own values is scarce. 

### 1.3. Job Insecurity and Its Consequences

In work and organizational psychology, job insecurity has been acknowledged as a predominant contemporary stress with which employees must cope [39]. Job insecurity is the perceived discrepancy between an employee’s experienced and preferred levels of employment security [40]. However, operationalization of job insecurity has varied. Although most researchers operationalize job insecurity as a subjective phenomenon that is highly dependent on the work context and the employees’ perception of it [41], others have operationalized job insecurity through the form of employment, such as holding a temporary contract [42,43]. Much research has focused on identifying potential consequences of job insecurity. These research efforts have been summarized in three meta-analyses [44,45,46] and several reviews [47,48,49,50,51,52]. This cumulative research shows that job insecurity is related to a variety of negative individual and organization-based outcomes [44,45,46]. Specifically, job insecurity is associated with individual outcomes such as low self-esteem [53] and high anxiety [54]. Moreover, job insecurity has also been associated with impaired work performance [45]. Effects that most likely develop over a longer time frame are associated with different aspects of psychological and physical health, such as burnout and depression [55], psychiatric problems [56], and high sickness presenteeism (i.e., attending work while ill [57]. Detrimental work-related outcomes due to job insecurity include low job satisfaction [58], diminished commitment [59], negatively affected organizational trust and loyalty [60,61], higher turnover intention, lower work performance, and greater incivility at work [62]. Within the framework of the JD-R model, job insecurity has been considered to be a job hindrance [63,64,65], which also reflects the overall findings of its impact on ill-being. In contrast to the large body of research within work and organizational psychology that examines health, studies of job insecurity among HPCs are relatively scarce. Olusoga and colleagues [9] found that job insecurity was a significant stressor for six elite coaches participating in their qualitative study. Similarly, using a mix-method approach, Singh [66] found that job insecurity was a stressor for most of their 25 participating South African professional coaches. Furthermore, job security was found to be related to greater psychological need satisfaction in a quantitative study among 418 voluntary and paid coaches [67]. Altogether, job insecurity also seems to be a relevant stressor among HPCs. However, the research is still limited. For instance, no studies have explored job insecurity longitudinally. Furthermore, research that examines how coaches’ perceptions of job insecurity are related to their psychological well- and ill-being is still lacking. 

Overall, research conducted within work and organizational psychology points to possible detrimental consequences of both value incongruence and job insecurity for employees’ psychological health. Thus, it seems plausible to examine more closely how job insecurity and value incongruence could act as stressors within the population of HPCs and potentially, over time, affect their psychological health at work. Hence, the overall aim of the current study was to explore how HPCs’ job insecurity and job value incongruence were related to their psychological well- and ill-being over time. On the basis of previous research, the hypothesis is that coaches’ perception of job insecurity and job value incongruence during the middle of the season would predict, respectively, coaches’ psychological well-being negatively and ill-being and positively, at the end of the season.

## 2. Materials and Methods

### Procedure and Participants

A longitudinal quantitative design was used, with HPC participants from a variety of sports in Norway and Sweden who responded to an online questionnaire at the start (T1), middle (T2), and end (T3) of their competitive seasons. Coaches were selected, based on their sports, to represent summer and winter, as well as individual and team sports (soccer 22.7%; individual winter sports (cross country skiing, biathlon, ski-jumping, alpine, speed skating) 28.4%; ice hockey 4.7%; track and field 12%; swimming 10%; orienteering 5%; handball 9.7%; volleyball 3.7%; and basketball 3.7%). Only coaches working with athletes who were competing at the highest national levels within their sport and in their country were included, thus, operationalizing HPC for the present study. Hence, for team sports, coaches for the highest competitive leagues were included, whereas for individual sports, coaches for athletes on national teams, recruiting teams, or elite clubs were included. The operationalization for selecting coaches for individual sports was done in collaboration with the respective sport federations. Response rates for each time point were the following: T1, *N* = 467 (54.7%); T2, *n* = 338 (39.6%); and T3, *n* = 343 (40.2%). All participants signed written informed consent prior to participating and the study was approved by the Norwegian Social Science Data Services (project number 26524) and the regional Ethical Review Board in Sweden (protocol number 2011/5:3). The study was conducted in accordance with the Declaration of Helsinki. Although most participants were male (91.6%), representation was relatively equal for the two nationalities (Norwegian 56.6%) and coaches of individual (44.5%) versus team sports. On average, the coaches were 40.7 years of age (SD = 9.93) and had 13.6 years (SD = 9.8) of coaching experience. Most coaches had a temporary contract 56.9% (*n* = 170), whereas 12.4% (*n* = 37) had a permanent contract, and 3% (*n* = 9) had no official contract. Additionally, 27.8% (*n* = 83) did not provide information about their type of contract.

## 3. Measures

### 3.1. Job Insecurity and Job Value Incongruence

The two variables, job insecurity and job value incongruence, were deliberately chosen to be measured at only T2 within the current study, as the aim was to explore the longitudinal consequences of experiencing job insecurity and job value incongruence. Professional experience mid-season was identified as the most information-rich time point measuring these two variables among HPC coaches as compared with the beginning or the end of season. At mid-season, coaches typically have developed a sense of how their values fit within their jobs. Additionally, based on coaches’ own perceptions of “meeting expectations”, perceived job insecurity is meaningful to measure at mid-season. To do this at the beginning of the season with very limited knowledge or at the end of the season with too much knowledge would confound the perception in very different ways. Most often, at the end of the season, coaches have come to terms with decisions regarding next season. 

Job security was measured with a single item: “How sure are you that you will have the same position next year?”, with response alternatives ranging from 1 (not sure at all) to 7 (completely sure). The was a reversed-scored item for analyses, and therefore higher values indicated greater job insecurity. This item reflects cognitive job insecurity and is an adapted item from the Job Insecurity Scale [68]. 

Coaches’ perceived job value incongruence was measured using the reverse-scored value subscale from the Areas of Worklife Scale [69]. Each of the five items (e.g., “my values and the organization’s values are alike”) were answered using a seven-point Likert-scale ranging from 1 (strongly disagree) to 7 (strongly agree). The subscale has previously shown acceptable internal consistency in the population of employees [70] and among athletes [71].

### 3.2. Psychological Well-Being and Ill-Being

The primary goal was to explore coaches’ psychological health at the end of the season. However, the purpose was also to study psychological health over time, thus, measures of psychological health were collected at both T1 and T3. Four measures reflecting symptoms of psychological well-being and ill-being were used. Psychological well-being was operationalized as vitality at work and satisfaction with work. The response format was a seven-point Likert scale ranging from 1 (strongly disagree) to 7 (strongly agree). Vitality at work was measured using the six-item Subjective Vitality Scale (e.g., “I feel alive and vital”) [72]. A five-item adapted version of the Satisfaction with Life Scale [73] was used to measure coaches’ satisfaction with work (e.g., “in most ways, my work is close to my ideal”). The scales have both previously shown good internal consistency when used in Norwegian populations [74,75]. Psychological ill-being was operationalized as emotional exhaustion and cynicism, the two most prominent subscales of the Maslach Burnout Inventory—General Scale [76]. Five items each were used, respectively, to measure both exhaustion (e.g., “I feel emotionally drained from my work”) and cynicism (e.g., “I have become less interested in my work since I started this job”). For each item, the response options included: 0 (never), 1 (a few times a year or less), 2 (once a month or less), 3 (a few times a month), 4 (once a week), 5 (a few times a week), and 6 (every day). The Norwegian version of the Maslach Burnout Inventory–General Scale has been validated both across occupational groups and over time [77].

## 4. Data Analysis

Data were screened using SPSS version 24 [78]. Little’s MCAR test indicated that data were missing at random (χ^2^ = 9436.14, df = 9276, *p* = 0.120). These data were determined to be normally distributed, as all items were within the +/− 3 range for skewness and 10 for kurtosis [79,80]. Structural equation modeling was conducted for all other analyses using Mplus (version 8.1) [81] using MLR estimation. Model fit was evaluated using combinations of the following goodness of fit indices [82,83]: comparative fit index (CFI) ≥ 0.90, Tucker–Lewis index (TLI) ≥ 0.90, standardized root mean square residual (SRMR) ≤ 0.08, and root mean square error of approximation (RMSEA) ≤ 0.08. Confirmatory factor analysis was used as a preliminary analysis to access the factor structure of the five latent constructs [84]. After finding an acceptable model fit for all latent variables [82], internal consistency for each scale was also explored with Cronbach’s alpha in SPSS [85]. Finally, two structural models with latent constructs were tested. In the first model, psychological well-being at T3 was predicted by job insecurity and job incongruence at T2, while controlling for psychological well-being at T1. In the second model, psychological ill-being at T3 was predicted by job insecurity and job incongruence at T2, while controlling for psychological ill-being at T1.

## 5. Results

### 5.1. Preliminary Results

All variables, except for cynicism at T1 and T3, showed acceptable model fit when exploring the CFA (see Table 1). For cynicism at T1 and T3, one item showed a lower-than-recommended parameter estimate (<0.3) [80] and therefore was dismissed from this latent variable. This particular item has previously indicated low parameter estimate [86]. For results of mean comparisons of study variables based on contract type, see Supplementary Materials. Correlation analyses indicated significant relations among all variables in the expected directions (see Table 2).

### 5.2. Main Results

**Figure 1:**
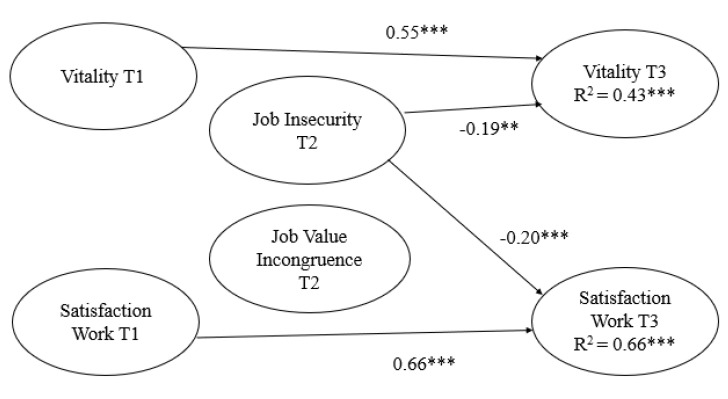
Job insecurity and job value incongruence at work predicting psychological well-being. *N* = 299. Model fit: χ^2^ (333) = 767.33, *p* = 0.000, CFI = 0.91, TLI = 0.90, RMSEA = 0.07 (90% CI 0.06–0.07), SRMR = 0.05; * *p* < 0.05, ** *p* < 0.01, *** *p* < 0.001.

**Figure 2:**
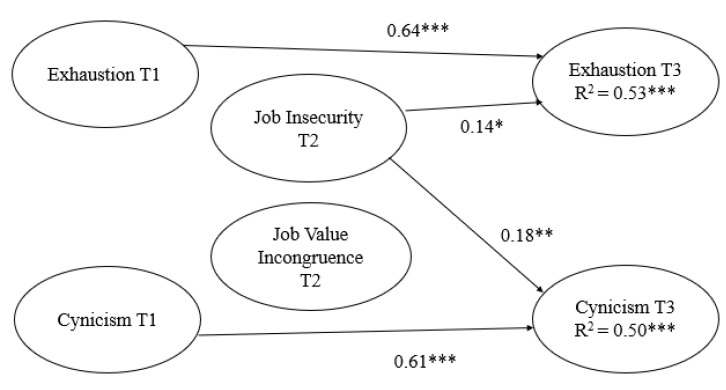
Job insecurity and job value incongruence predicting psychological ill-being. *N* = 299. Model fit: χ^2^ (233) = 480.75, *p* = 0.000, CFI = 0.91, TLI = 0.90, RMSEA = 0.06 (90% CI 0.05–0.07), SRMR = 0.06. * *p* < 0.05, ** *p* < 0.01, *** *p* < 0.001.

Model 1, investigating psychological well-being as the outcome, showed acceptable data fit (see Figure 1). Forty-three percent of the variance in vitality at T3 and 66% of the variance in satisfaction with work at T3 were explained. In addition, job insecurity at T2 negatively predicted changes in both vitality (ß = −0.19) and satisfaction with work (ß = −0.20) at T3, after controlling for vitality (ß = 0.55) and satisfaction with work (ß = 0.66), respectively, at T1. Contrary to the hypothesis, job value incongruence at T2 did not significantly predict either variable representing change in psychological well-being at T3. Model 2, investigating psychological ill-being as the outcome, also showed acceptable data fit (see Figure 2). Fifty-three percent of the variance in exhaustion at T3 and 50% of the variance in cynicism at T3 were explained. Job insecurity at T2 positively predicted both change in exhaustion at T3 (ß = 0.14) and cynicism at T3 (ß = 0.18), after controlling for exhaustion at T1 (ß = 0.64) and cynicism at T1 (ß = 0.61), respectively. Contrary to the hypothesis, job value incongruence at T2 did not significantly predict either of the variables representing change in psychological ill-being at T3.

## 6. Discussion

The overall findings indicated that coaches who experienced higher levels of job insecurity during the middle of their season reported lower levels of psychological well-being, and higher levels of psychological ill-being, at the end of their season. In contrast to research findings in the general work and organizational psychology literature [44,45,46], job value incongruence during the middle of the season did not predict either psychological well- or ill-being at the end of the season for the coaches.

### 6.1. Values: A Contributor to Sustainability in the Coaching Profession?

Although psychological well- and ill-being was unaffected by job value (in)congruence over time when job insecurity was included in the model, it is noteworthy that the correlational results between job value incongruence (T2) and psychological well- and ill-being (T3), nonetheless, showed a significant association in the proposed direction. These results are somewhat unexpected when compared with a number of studies that reported in the general occupational domain [37,87], and with the limited research on coaches in sports [34,38]. Therefore, we critically discuss the findings as a possible consequence of a weak theoretical link between how value is conceptualized in sport science as compared with models developed within work and organizational psychology such as the JDR model, and consequently, also including methodological limitations associated with conceptualization. 

First, the theoretical conceptualization of “value” in HP sport needs explicit attention, particularly how the role of values influences coaching behavior in practice and their psychological health. Thus far, two very different approaches to values have been explored in sport science, and both differ considerably from the approach used in the JDR model within organizational psychology. One approach was based on coaching philosophy, a broad and poorly defined construct that has been used in coach education and in the coaching literature for many decades [88], which included “value” as a limited and integrated aspect. Thus, understanding values for a coach in relation to the more complex term, coach philosophy, was essential. The other approach that addressed values was the Acceptance and Commitment Therapy (ACT), recently applied to sport science and practice [89,90]. Briefly, the purpose of ACT is to increase psychological flexibility, simply meaning the ability to be open and present, and do what is important in life. Within ACT, there is a strong emphasis on values and how they are connected to behaviors that increase quality of life and health. We argue that future research should take up this approach when further exploring whether value incongruence is a contributor to limited sustainability and poor mental health in the coaching profession. 

Second, psychometrically sound measurements need to be adapted for coaches of HP sport, based upon a clear conceptualization of value. Items developed and used for more traditional occupations (i.e., the measurement of Leiter and Maslach) [69] may not apply in the HP sports context. Moreover, the best way to capture person-environment fit [91] is an ongoing debate amongst researchers. Person-environment fit (e.g., congruence between personal and organizational values) can be operationalized as a perceived fit measure as used herein. However, this type of operationalization has been criticized due to its high correlation with job attitudes. This has made researchers question the distinctiveness of these constructs [92]. Researchers within occupational psychology in sport should engage in this debate in future research, to better understand how person-environment fit could be better operationalized and measured when it comes to coaches’ value incongruence at work. 

### 6.2. The Role of Job Insecurity in HP Sport

Working as a coach in the context of HP sport is characterized by HP demands, short-term contracts, and a risk of being fired. Thus, job insecurity is a normalized, integral part of this profession [93]. Consequently, HPC research has paid attention to stress, burnout, and mental health [5] but job insecurity has received limited attention. A recent study from Spain, including a large coach population (*N* = 1481) representing different levels, found that insecurity over working conditions and emotional demands was significantly elevated as compared with reference values of the Spanish general work force [94]. In contrast, the general occupational health research has focused on both the consequences and preventive strategies of job insecurity in order to decrease mental health problems among employees [48]. The current study contributes by exploring and critically discussing how job insecurity compares across the HPC context to a more traditional occupational health context, in which white- and blue-collar employees have been primarily investigated [45,49]. Typically, HPC have temporary contracts that are often strongly related to performance outcomes (i.e., win-loss records) [95,96]. In practice, unsatisfactory performance can result in the termination of the employment contract, sometimes during the season [96,97]. Indeed, performance outcomes are important for employees in other sectors, however, the consequences are not equally dramatic and/or other employees have more strongly protected positions (e.g., through support from trade unions). In general, most employees have the expectation of a safe and secure position, in contrast to HPCs who work under a constant threat of losing their jobs because of failure [98]. Interestingly, research that focused on occupational health has established that employees holding temporary contracts are naturally more insecure about their jobs as compared with permanent employees [99]. However, findings have been mixed regarding the associations between job insecurity among temporary employees and negative outcomes, while consequences that have been identified were only moderate [47,100,101]. This can be explained by the psychological contract theory [102], i.e., for temporary employees, job security may not be included in their psychological contract, the unspoken expectations employees have of their employers. When experiencing job insecurity, the psychological contract of temporary employees is unbroken, explaining why consequences have been less negative. For HPCs, job insecurity is an integral, expected part of their job. However, our results show that over time, HPCs still experience reduced well-being and increased ill-being. Employability, i.e., the employee’s assessment that it is possible to find another equivalent job [103], has been discussed as an important buffer to job insecurity [102]. However, it could be difficult for HPCs to find comparable jobs since positions in their field are limited, and hence employability might not act as a buffer for them. Taken together, questions remain regarding how HPCs react to job insecurity and how these potential effects can be buffered.

### 6.3. Practical Implications

Job insecurity will always be a challenge within the HP sport setting. In addition, it can be argued that it is both unprofessional and unethical to place responsibility for success solely on individual coaches, regardless of their awareness or acceptance of the competitive sport culture. A more humanistic and sustainable approach would be to fully acknowledge these issues and provide support to manage job insecurity challenges and, when appropriate, also provide mental health support. The most comprehensive approach would involve all stakeholders, including employers within organizations, national governing bodies, and coach education. Regarding educational programs for coaches, the topic of job insecurity needs appropriate attention, and adaptive strategies should be emphasized. For example, coaches should discuss the competitive hiring process, what standard job contracts look like, the risks of being fired, strategies for communicating about job insecurity with one’s employer, and coping strategies during challenging periods (e.g., at the end of a contract period or when one has been fired). Importantly, it is ultimately the responsibility of the employer to bring the topic of coping with job insecurity to the table. Employers must provide professional guidelines and systematically develop strategies in order to manage their employees who work in an insecure environment. For example, they need to take timely initiative regarding potential contract renewal or termination, before an existing contract ends. They should also maintain ongoing communication about, and support surrounding, job security and mental health when pressures build. Moreover, we argue that an increased organizational awareness about coaches’ job insecurity is needed to advance this topic in practice. It would be a first step if organizations would reflect on and recognize the benefits that are associated with improved job security for coaches. Ill-being may be associated with turnover, lack of retention, loss of experience, and the challenge and cost of replacement. It is our hope that organizational-level leaders will acknowledge how enhanced job security can be associated with improved mental health, and with more motivated and efficient coaches. Finally, we argue that paying appropriate and consistent attention to job insecurity has the potential to benefit all participants in the HP sport community.

### 6.4. Limitations and Future Research

There are a number of limitations to our measurement of the study concepts. First, a single-item measure of job insecurity was used, of which the psychometric properties cannot be determined, making it more difficult to capture this multifaceted construct [104]. However, the single item does capture the respondent’s likelihood of keeping their current job, which represents the core of cognitive job insecurity [105]. Furthermore, a meta-analysis has indicated that single-item measurements tend to underestimate the relationship between job insecurity and potential outcome [46]. In addition, herein, job insecurity only concerned the likelihood of potential job loss (cognitive job insecurity) [40]. Future studies should, therefore, include the affective component, i.e., worry related to a potential job loss, with which stronger relations to outcomes have been found [105]. Moreover, through this operationalization of job insecurity it cannot be determined how the (in)voluntary nature of the employment insecurity may affect the outcome. As job insecurity seems to be important and linked to well-being in HPC, future research should make use of other kinds of measures for job insecurity that allow for a better differentiation of the different subforms of job insecurity [68,106,107]. Secondly, future studies should investigate HPC value (in)congruence using a theoretical approach adapted for, and meaningful to, the sport-specific context. This implies using a measure matched to the HPC context, as well as considering a calculated fit measure [92]. We argue that the construct of job insecurity within HPC is in its infancy, especially as compared with the attention it has been given within traditional occupations. Thirdly, we argue that future studies should measure both job insecurity and value incongruence over several time points to explore how fluctuations in these variables affect psychological health. Moreover, we acknowledge that the four measures used to represent mental health in the current study do not grasp the complexity of the concept. Hence, the population of HP coaches would benefit from comprehensive future research and practices addressing this topic, with the aim of better managing mental health and retention in a typically vulnerable profession. First, job insecurity should be explored within a larger scope, in which sustainability of the HP context is linked to both mental health and performance for all participants (i.e., athletes and coaches). Second, since job insecurity will always be a part of HP sport, how HP coaches cope with this uncertainty needs to be further explored. Finally, we suggest that the topics of job insecurity and value incongruence in relation to psychological health should also be explored in future qualitative studies to deepen our understanding of nuances in such experiences among HP coaches. Such research findings could provide a better understanding of more flexible ways to handle this situation, from the perspectives of individual coaches, as well as organizations and employers. In addition, we need to understand how to implement the topics of job insecurity, career awareness, and career transitions within educational programs for coaches [108,109].

## 7. Conclusions

Job insecurity is an expected and integral part of HPC. Nevertheless, it can be concluded that similar to findings in occupations outside of sports, coaches’ perceptions of job insecurity matter to their psychological health at work. In order to give more attention to sustainability and mental health in the coaching profession, stakeholders and employers should consider investing time and resources to advance this challenging topic, both in practice and in research. In contrast to coherent findings in work and organizational psychology, the role of values and job value incongruence in HPC is still poorly understood and needs more attention. The first step should be to develop a strong theoretical conceptualization of value in HP sport. This should include how the role of values influences coaching behavior and psychological health. Finally, it is argued that greater knowledge about job insecurity and values in HPC would provide better opportunities to develop and implement a best practice model. Ultimately, this could make the demanding profession more sustainable and attract greater diversity of coaches at the HP level. 

## Figures and Tables

**Table 1:** Confirmatory factor analysis and alpha values.

Variable	χ^2^ (fd)	CFI	TLI	RMSEA (90% CI)	SRMR	Alpha
Value Incongruence T2	7.05 (4)	0.99	0.98	0.05 (0.00–0.11)	0.02	0.79
Exhaustion T1	6.42 (4)	0.99	0.98	0.05 (0.00–0.11)	0.02	0.84
Exhaustion T3	1.84 (3)	1.00	1.00	0.00 (0.00–0.08)	0.01	0.89
Cynicism T1	2.70 (1)	0.98	0.90	0.08 (0.00–0.19)	0.01	0.71
Cynicism T3	0.16 (1)	1.00	1.00	0.00 (0.00–0.11)	0.00	0.83
Vitality T1	20.99 (8) *	0.98	0.97	0.07 (0.04–0.11	0.03	0.91
Vitality T3	15.19 (6) *	0.99	0.98	0.07 (0.03–0.12)	0.01	0.94
Satisfaction Work T1	4.95 (4)	1.00	1.00	0.03 (0.00–0.10)	0.02	0.80
Satisfaction Work T3	12.37 (5) *	0.98	0.96	0.07 (0.02–0.12)	0.03	0.83

* *p* < 0.05; χ^2^ = Chi-square; df, degrees of freedom; CFI, comparative fit index; TLI, Tucker-Lewis index; RMSEA, root mean square error of approximation and 90% confidence interval; SRMR, standardized root mean square residual.

**Table 2:** Correlation matrix among variables.

Variable	*M*	*SD*	1	2	3	4	5	6	7	8	9
1. Job Insecurity T2	2.92	1.92	-								
2. Value Incongruence T2	3.44	0.81	0.24 **	-							
3. Exhaustion T1	1.69	1.06	0.19 **	0.13 *	-						
4. Exhaustion T3	1.90	1.22	0.31 **	0.23 **	0.65 **	-					
5. Cynicism T1	0.94	0.97	20 **	0.19 **	0.44 **	0.33 **	-				
6. Cynicism T3	1.29	1.25	32 **	0.26 **	0.38 **	0.62 **	0.56 **	-			
7. Vitality T1	5.30	1.07	−0.18 **	−0.23 **	−0.51 **	−0.47 **	−0.42 **	−0.47 **	-		
8. Vitality T3	4.93	1.31	−0.33 **	−0.27 **	−0.39 **	−0.64 **	−0.29 **	−0.61 **	0.58 **	-	
9. Satisfaction Work T1	4.87	1.04	−0.31 **	−0.32 **	−0.32 **	−0.37 **	−0.37 **	−0.43 **	0.48 **	0.40 **	-
10. Satisfaction Work T3	4.65	1.21	−0.41 **	−0.33 **	−0.30 **	−0.50 **	−0.27 **	−0.57 **	0.40 **	0.59 **	0.68 **

* *p* < 0.05; ** *p* < 0.01; SPSS 24 was used to calculate means and standard deviations; reported means of latent variables are zero in cross-sectional analyses. Correlations between variables such as job insecurity were only measured with one construct (i.e., not a latent variable).

## Supplementary Materials

The following are available online at https://www.mdpi.com/1660-4601/17/19/6939/s1, Table S1. One-way ANOVA for differences in types of coaching contracts related to study variables.
