# Supplementary material for: Impact of Job Insecurity on Psychological Well- and Ill-Being among High Performance Coaches
Source: Int J Environ Res Public Health. 2020 Sep 23;17(19):6939. doi: 10.3390/ijerph17196939 (PMC7579261; doi:10.3390/ijerph17196939)
Supplement: Supplementary file 1 [file ijerph-17-06939-s001.pdf]

International Journal of
Environmental Research and Public Health

MDPI

Table S1. One-way ANOVA for differences in types of coaching contracts related to study variables.

**ANOVA**	Group 1 Permanent Position	Group 2 Fixed Position	Group 3 Not specified Position	Variance		*p*
**Variable**	M (SD)	M (SD)	M (SD)	F	df	
Value T2	3.30 (0.75)	3.45 (0.81)	3.49 (0.84)	0.732	2, 287	0.482
Job insecurity T2	2.20 (1.58)	3.03 (1.92)	3.02 (2.00)	3.134	2, 292	0.045[a]
Exhaustion T1	1.82 (1.17)	1.64 (1.06)	1.73 (1.00)	0.548	2, 290	0.579
Exhaustion T3	1.89 (1.21)	1.83 (1.22)	2.04 (1.23)	.848	2, 291	0.429
Cynicism T1	1.27 (1.13)	0.91 (0.93)	0.85 (0.94)	2.667	2, 286	0.071
Cynicism T3	1.39 (1.37)	1.24 (1.21)	1.34 (1.29)	0.280	2, 287	0.756
Vitality T1	5.18 (1.30)	5.40 (1.09)	5.18 (.91)	1.606	2, 292	0.203
Vitality T3	4.94 (1.60)	4.98 (1.27)	4.85 (1.26)	0.264	2, 293	0.768
Satisfaction with work T1	4.73 (1.12)	4.96 (1.02)	4.78 (1.04)	1.213	2, 291	0.299
Satisfaction with work T3	4.82 (1.29)	4.67 (1.93)	4.53 (1.20)	0.816	2, 292	0.433

Group 1, n range = 36–37; Group 2, n range = 161–169; Group 3, n range = 88–92; [a] significant difference when multiple comparison with Tuckey post hoc test between Group 1 and Group 2, $p \leq 0.05$.
